# Cabozantinib, an Anti-Aging Agent, Prevents Bone Loss in Estrogen-Deficient Mice by Suppressing Senescence-Associated Secretory Phenotype Factors

**DOI:** 10.3390/ijms26157123

**Published:** 2025-07-24

**Authors:** Jueun Lee, Jiin Oh, Jae-Ryong Kim, Hyunil Ha, Taesoo Kim, Daewon Jeong

**Affiliations:** 1 Department of Microbiology, Laboratory of Bone Metabolism and Control, Yeungnam University College of Medicine, Daegu 42415, Republic of Korea; wndms4864@ynu.ac.kr (J.L.); jjvmf@ynu.ac.kr (J.O.); 2 Department of Biochemistry and Molecular Biology, Senotherapy-Based Metabolic Disease Control Research Center, Yeungnam University College of Medicine, Daegu 42415, Republic of Korea; kimjr@med.yu.ac.kr; 3 Herbal Medicine Research Division, Korea Institute of Oriental Medicine, Daejeon 34054, Republic of Korea; hyunil74@kiom.re.kr (H.H.); xotn91@kiom.re.kr (T.K.)

**Keywords:** senescent cell, SASP, anti-aging agent, cabozantinib, osteoblast and osteoclast differentiation, ovariectomy, anti-osteoporotic agent

## Abstract

Senescent cells secrete pro-inflammatory cytokines, collectively referred to as the senescence-associated secretory phenotype (SASP). Certain pro-inflammatory SASP factors are known to inhibit the differentiation of bone-forming osteoblast while promoting the differentiation of bone-resorbing osteoclasts, thereby causing osteoporosis. In this study, we screened cabozantinib, a tyrosine kinase inhibitor used to treat medullary thyroid cancer, for its ability to reduce doxorubicin-induced cellular senescence in both osteoblast and osteoclast progenitors. This non-cytotoxic agent suppressed the secretion of SASP factors (e.g., TNFα, IL1α, IL1β, IL6, and CCL2) from senescent osteoblast and osteoclast progenitors, resulting in enhanced osteoblast differentiation and reduced osteoclast differentiation. Furthermore, intraperitoneal administration of cabozantinib to age-related estrogen-deficient mice subjected to ovariectomy prevented bone loss without apparent side effects, increasing osteoblast numbers and reducing osteoclast numbers along the surface of the trabecular bone. In summary, our findings suggest that anti-aging cabozantinib has potential as a preventive anti-osteoporotic agent by promoting osteogenesis and inhibiting osteoclastogenesis through the repression of SASP.

## 1. Introduction

Aging is a continuous and irreversible pathophysiological process characterized by functional decline across the entire body, including cells, tissues, and organs. This gradual deterioration leads to a higher incidence of age-related diseases, such as neurodegenerative disorders, including Alzheimer’s disease, Parkinson’s disease, Huntington’s disease, and depression, as well as other conditions like osteoporosis, myocardial ischemia and reperfusion, atherosclerosis, and diabetes [1,2]. Cellular senescence is triggered by several inducers, including telomere shortening, DNA damage, chromatin alterations, reactive oxygen species, and oncogene activators [3,4]. Senescent cells exhibit an abnormal, enlarged, and flattened morphology with ruffled surfaces [5], and are defined by a stable, irreversible arrest of the cell cycle mediated via two signaling pathways of ataxia telangiectasia mutated (ATM)/p53/p21^CIP1^ and p16^INK4a^/retinoblastoma (RB) [6]. Despite this arrest, senescent cells remain metabolically active. ATM-mediated phosphorylation of p53 stabilizes the protein and induced expression of the cyclin-dependent kinase inhibitor p21^CIP1^, thereby promoting G1-phase cell cycle arrest [7]. Together, p16I^NK4a^ inhibits CDK4 and CDK6, suppressing RB phosphorylation and maintaining RB in a hypophosphorylated state, which blocks cell cycle progression from the G1 to S phase [8]. As the cellular micro-environment changes with age, senescent cells begin to secrete senescence-associated secretory phenotypes (SASPs) factors. These include pro-inflammatory cytokines [e.g., interleukin (IL)-1α, IL-1β, IL-6, and IL8], chemokines [e.g., C-C motif ligand 1 (CCL1), CCL2, and CCL5], proteases (e.g., matrix metalloprotease and serine protease), and growth factors (e.g., PDGF) [9,10]. SASP factors exhibit dual roles [6]: they contribute to tissue regeneration, tumor suppression, and immunosurveillance, but can promote inflammation, tissue damage, and cancer progression. Consequently, extensive research has focused on anti-aging strategies that target senescent cells.

Bone homeostasis is maintained by a delicate balance between bone-forming osteoblasts and bone-resorbing osteoclasts [11]. With aging, particularly post-menopause, this balance is disrupted, leading to impaired bone formation and increased resorption, thereby increasing the risk of osteoporosis and fractures [12,13]. In aging bone, mesenchymal stem cells are more likely to differentiate into adipocytes rather than osteoblasts [14,15]. Moreover, SASP factors such as tumor necrosis factor-alpha (TNFα), IL1α, IL1β, IL6, and CCL2 are secreted by senescent cells, fostering a pro-inflammatory microenvironment within bone tissue [16]. TNFα, IL1α, and IL6 specifically impair osteoblast differentiation and enhance osteoclastic bone resorption [17,18]. These findings highlight the importance of therapeutic strategies targeting senescent cells (senolytics) or modulating SASP activity (senomorphics) to prevent age-related osteoporosis.

In this study, we screened cabozantinib, a tyrosine kinase inhibitor approved for medullary thyroid cancer [19], for its anti-aging effects in bone-related cells, specifically osteoblasts and osteoclasts. Cabozantinib demonstrated the ability to activate osteoblasts and inhibit osteoclasts by suppressing the secretion of SASP factors from these cells. Additionally, it prevented bone loss in estrogen-deficient, ovariectomized mice. Our findings indicate that targeting senescent osteoblastic and osteoclastic cells using cabozantinib could be a potential therapeutic approach for treating age-related osteoporosis.

## 2. Results and Discussion

### 2.1. Cabozantinib Blocks Senescence in Osteoblast and Osteoclast Progenitors

**Figure 1:**
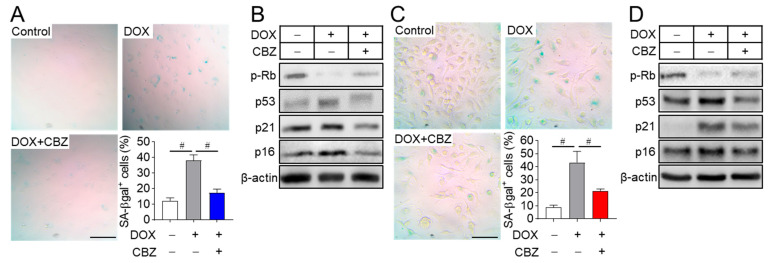
Cabozantinib prevents cellular senescence. Osteoblast (**A**) and osteoclast progenitors (**C**) were incubated with 0.2 μM and 1 µM doxorubicin (DOX) for 4 h to induce cellular senescence, followed by treatment with 100 nM cabozantinib (CBZ) for 6 and 4 days, respectively. Cells were stained for SA-β-gal, and images were captured from four randomly selected fields under a microscope. The graph shows the percentage (%) of SA-β-gal-positive cells relative to the total number of cells, averaged from triplicate samples. Scale bar, 100 μm. To evaluate the expression of senescence markers, senescent osteoblast (**B**) and osteoclast progenitors (**D**) were treated with 100 nM CBZ for 24 h and 48 h, respectively. Cell lysates were then immunoblotted for phospho-Rb, p53, p21, and p16. β-actin served as the loading control. Scale bar, 100 μm. Data are presented as mean ± SD. # *p* < 0.01.

We aimed to identify agents with anti-aging properties from existing drugs and various natural products using a doxorubicin-induced senescent fibroblast model. Among the candidates screened, cabozantinib, an anticancer drug approved for medullary thyroid cancer [19], emerged as one of the most effective compounds in preventing cellular senescence. To assess its applicability in in vitro bone-related cell experiments, osteoblast and osteoclast progenitors were treated with cabozantinib across a range of concentrations based on the primary screening. The optimal non-cytotoxic concentration for naive osteoblast and osteoclast progenitors was determined to be 100 nM. This concentration also had no impact on the viability of doxorubicin-induced senescent osteoblast and osteoclast progenitors (Figure S1) and reduced the number of senescent cells with enlarged and flattened morphology (Figure 1A,C). To further evaluate the effect of cabozantinib on cellular senescence, senescent osteoblast and osteoclast progenitors were treated with cabozantinib and stained using senescence-associated β-galactosidase (SA-β-Gal), a widely recognized marker of replicative senescence in mammalian cells [20]. While SA-β-Gal-stained activity was significantly increased in senescent osteoblast and osteoclast progenitors compared to non-senescent cells (control), cabozantinib effectively inhibited the progression to the senescent cells (Figure 1A,C). Cellular senescence is known to involve irreversible proliferative arrest mediated by the p53/p21 and p16/Rb tumor suppressor pathways [8,21,22]. In this study, the elevated activation of p53, p21, and p16, along with hypo-phosphorylation of RB in senescent cells, was reversed to basal levels observed in non-senescent control cells upon cabozantinib treatment (Figure 1B,D). These findings suggest that cabozantinib exerts anti-aging effects by repressing the p53/p21 and p16/Rb-mediated senescence pathways.

### 2.2. Cabozantinib Promotes Osteoblast Differentiation and Inhibits Osteoclast Differentiation by Repressing SASP

**Figure 2:**
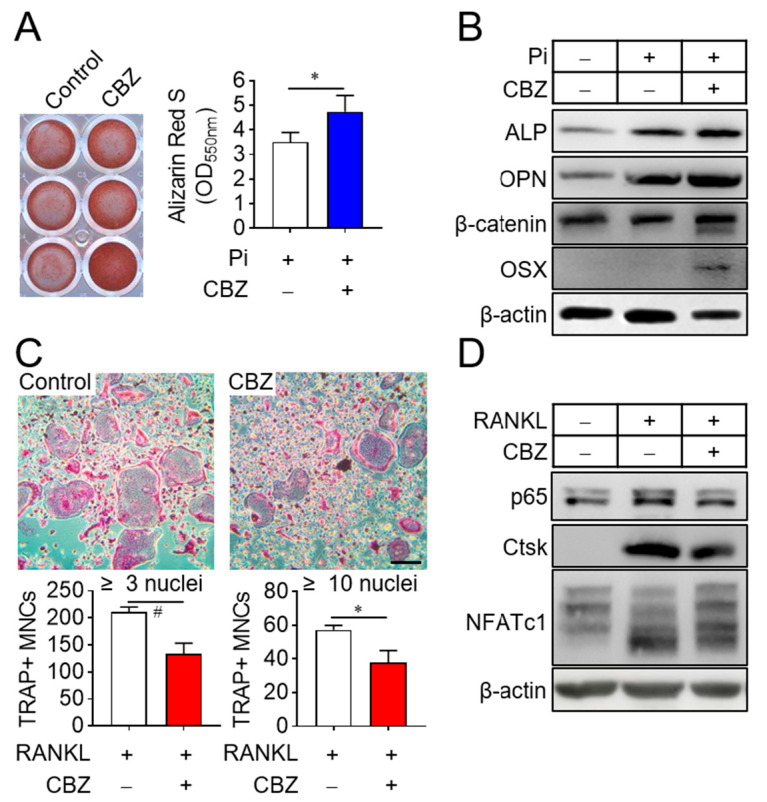
CBZ stimulates osteoblast differentiation and inhibits osteoclast differentiation. (**A**) Osteoblast differentiation: Senescent osteoblast progenitors were cultured in osteogenic media containing 4 mM phosphate with or without 100 nM CBZ for 10 days. Cells were stained with alizarin red S, and images were acquired by scanning the plate. The dye was solubilized using cetylpyridinium chloride, and absorbance at 550 nm was measured to quantify calcium deposition. (**B**) Osteogenic gene expression: Osteoblast progenitors were subjected to the indicated treatments, and cell lysates were collected on day 8 of differentiation for immunoblotting with antibodies against ALP, OPN, β-catenin, and OSX. (**C**) Osteoclast differentiation: Senescent osteoclast progenitors were differentiated into multinucleated osteoclasts in the presence of M-CSF (30 ng/mL) and RANKL (100 ng/mL) in the absence or presence of 100 nM CBZ, for 4 days. TRAP staining was used to identify TRAP-positive multinucleated cells [TRAP(+) MNCs] containing more than 3 or 10 nuclei, which were counted under a microscope. (**D**) Osteoclastogenic gene expression: After the indicated treatments, osteoclast progenitor lysates were collected on day 4 of differentiation and analyzed via immunoblotting for NFκB p65, Ctsk, and NFATc1. Scale bar, 100 μm. Data are expressed as mean ± SD. * *p* < 0.05; # *p* < 0.01.

Since bone remodeling is regulated by osteoclast-mediated bone resorption followed by osteoblast-mediated bone formation [23], we investigated the effects of cabozantinib on osteoblast and osteoclast differentiation. Treatment of senescent osteoblast progenitors with cabozantinib in osteogenic media supplemented with 4 mM inorganic phosphate enhanced osteoblast differentiation compared to untreated senescent cells (Figure 2A). This effect correlated with increased expression of osteogenic marker genes, including alkaline phosphatase (ALP), osteopontin (OPN), β-catenin, and osterix (OSX) [24] (Figure 2B). In contrast, cabozantinib suppressed the differentiation of senescent osteoclast progenitors into multinucleated osteoclasts in the presence of M-CSF and RANKL (Figure 2C). This inhibition was assessed by counting TRAP-positive multinucleated cells [TRAP(+) MNCs] with more than 3 or 10 nuclei and further supported by a reduced expression of osteoclastogenic marker genes such as NF-κB p65, cathepsin K (Ctsk), and NFATc1 [25] (Figure 2D). These results indicate that CBZ enhances osteoblast differentiation while inhibiting osteoclast differentiation under senescent conditions.

**Figure 3:**
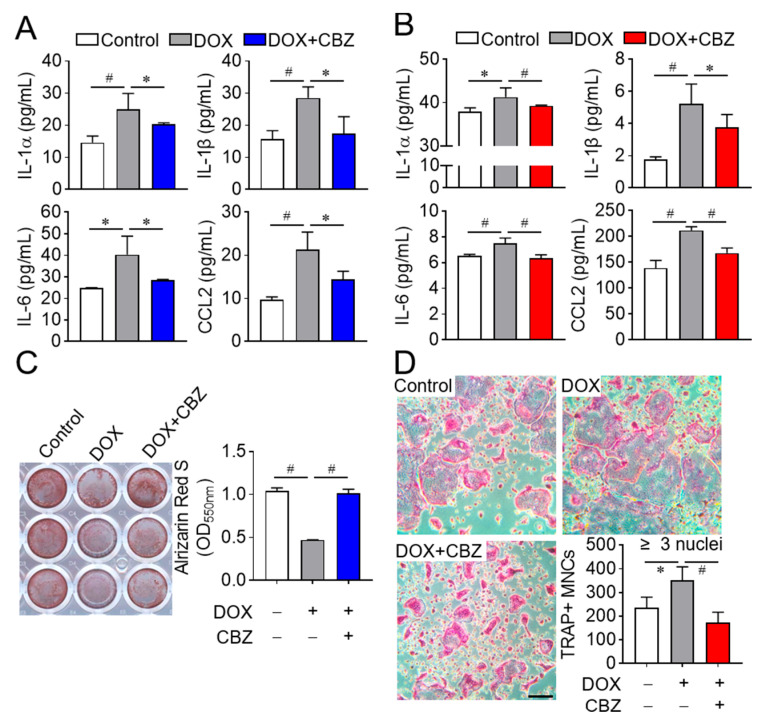
CBZ-mediated SASP repression enhances osteoblast differentiation and inhibits osteoclast differentiation. (**A**,**B**) ELISA for SASP factors. Osteoblast and osteoclast progenitors were cultured without or with doxorubicin, followed by further incubation in the absence or presence of CBZ (100 nM) for 4 days and 6 days, respectively. Culture media from osteoblasts (**A**) and osteoclast progenitors (**B**) were collected under the same conditions. The levels of SASP factors (TNF-α, IL-1α, ILβ, IL6, and CCL2) were quantified using ELISA. (**C**,**D**) Osteoblast and osteoclast differentiation. As in (**A**,**B**), conditioned media from control, doxorubicin (DOX), and DOX + CBZ (DOX+CBZ) were mixed 1:1 with fresh media, and used for differentiation of osteoblasts (**C**) and osteoclasts (**D**). Differentiated cells were stained with alizarin S and TRAP, respectively, and analyzed microscopically. Scale bar, 100 μm. Data are presented as mean ± SD. * *p* < 0.05; # *p* < 0.01.

Certain SASP factors secreted by senescent cells are known to inhibit osteoblast differentiation and stimulate osteoclast differentiation, contributing to bone loss resembling osteoporosis [26]. To determine whether SASP regulation accounts for the observed effects, we analyzed the levels of SASP factors (e.g., TNFα, IL1α, IL1β, IL6, and CCL2) in the culture media of non-senescent, senescent, and cabozantinib-treated senescent cells. Elevated levels of IL1α, IL1β, IL6, and CCL2 in senescent osteoblast and osteoclast progenitor media were reduced by cabozantinib, while TNFα remained undetectable (Figure 3A,B). To investigate whether these reductions in SASP factors affect cellular differentiation, we performed differentiation assays using conditioned media. Media from cabozantinib-treated senescent osteoblast progenitors increased osteoblast differentiation compared to media from senescent untreated senescent cells (Figure 3C). Conversely, media from cabozantinib-treated senescent osteoclast progenitors decreased osteoclast differentiation relative to their senescent counterparts (Figure 3D). Collectively, these results suggest that cabozantinib attenuates SASP secretion from senescent osteoblast and osteoclast progenitors, thereby promoting osteoblast differentiation and suppressing osteoclast differentiation.

### 2.3. Cabozantinib Restores Deteriorated Trabecular and Cortical Bone Microarchitecture

Previous studies have shown that senescence of bone marrow-derived mesenchymal stem cells contributes to ovariectomized (OVX)-induced osteoporosis, [27] and deletion of the senescent-related gene p16 rescues bone loss in OVX-mice [28], highlighting the critical role of senescence in estrogen deficiency-induced osteoporosis.

**Figure 4:**
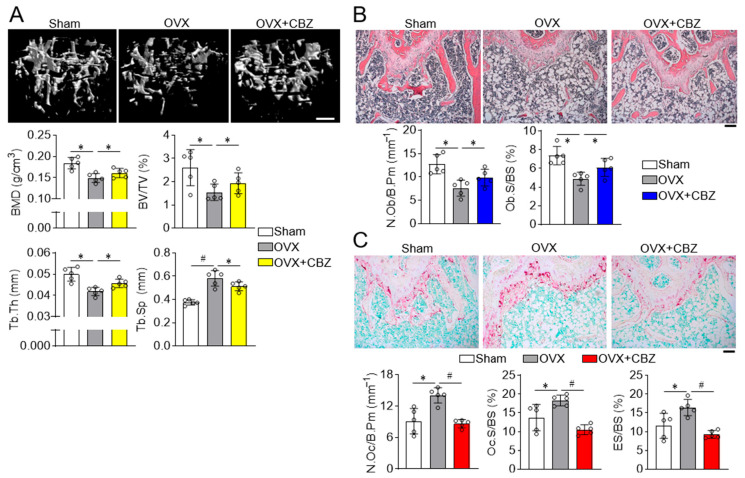
CBZ prevents bone loss in ovariectomized mice. (**A**) µCT analysis: Mice underwent either a sham operation or bilateral ovariectomy (OVX). CBZ (0.25 mg/kg; 500 nM) was administered intraperitoneally every two days for 8 weeks. µCT analysis of femurs was performed to assess bone mineral density (BMD), trabecular bone volume per total tissue volume (BV/TV), trabecular bone thickness (Tb.Th), trabecular bone separation (Tb.Sp). Scale bar, 0.5 mm. Osteoblast analysis: Longitudinal femur sections were stained for H&E to detect osteoblasts (**B**) and TRAP to analyze osteoclasts (**C**). NOb/B.Pm, number of osteoblasts per bone perimeter; Ob.S/BS, osteoblast surface per bone surface; NOc/B.Pm, number of osteoclasts per bone perimeter; Oc.S/BS, osteoclast surface per bone surface. Also, TRAP-stained sections were used to determine the eroded bone surface per bone surface (ES/BS). Scale bar, 100 μm. Data are presented as mean ± SD (*n* = 5 per group). * *p* < 0.05; # *p* < 0.01.

Given that cabozantinib enhances osteoblast differentiation and inhibits osteoclast differentiation in vitro, we next evaluated its ability to restore bone quality in an in vivo model of age-related, estrogen-deficient bone loss. Bone histomorphometry was evaluated using high-resolution Dual-energy X-ray Absorptiometry (DXA) and microcomputed tomography (µCT). While ovariectomized mice exhibited increased fat and body weight compared to sham-operated mice, cabozantinib treatment did not affect these parameters (Figure S2A). A whole-body DXA scan revealed that cabozantinib restored bone density and mineral content in ovariectomized mice (Figure S2B). Further µCT analysis demonstrated that cabozantinib significantly improved various trabecular bone indices, including bone mineral density, bone volume per total volume, thickness, and separation (Figure 4A), which was consistent with improvements obtained in cortical bone parameters (Figure S3).

Histological analysis of femur bone sections revealed that cabozantinib prevented the loss of H&E-stained osteoblasts on the trabecular surface (Figure 4B) and normalized the increased number of TRAP-positive osteoclasts observed in ovariectomized mice (Figure 4C). Additionally, cabozantinib reduced trabecular bone erosion adjacent to the bone marrow, suggesting decreased bone resorption. These results collectively indicate that cabozantinib has dual effects: enhancing osteoblast function and suppressing osteoclast activity, thereby exerting anti-osteoporotic efficacy in ovariectomized.

Cabozantinib is a tyrosine kinase inhibitor known to suppress tumor angiogenesis and metastasis [29]. In the present study, it demonstrated protective effects against osteoporosis when administrated at the onset of estrogen deficiency following ovariectomy. However, administration two months post-ovariectomy failed to suppress osteoporosis (Figure S4), indicating that cabozantinib is effective during the early stages of age-rewalted bone loss, but not in chronic osteoporosis. The overall results indicated that cabozantinib acts as a preventive agent but not as a therapeutic agent.

Clinically, cabozantinib is approved for renal cell carcinoma at a dosage of 60 mg per day and for progressive metastatic medullary thyroid cancer at 140 mg per day [30]. At these doses, it is associated with side effects such as diarrhea, palmar-plantar erythrodysesthesia, fatigue, hypertension, stomatitis, hypocalcemia, and proteinuria [31]. In a pre-clinical ovariectomized mouse model, the dose of cabozantinib was determined to be within an appropriate range between vitro concentrations and clinically approved doses as an anticancer drug. Notably, the effective dose of cabozantinib for anti-aging applications in osteoporosis is 0.25 μg/kg, which is four to nine times lower than its clinically approved doses, suggesting a reduced risk of adverse effects. In conclusion, we demonstrate that cabozantinib functions as a senolytic agent by modulating the p53/p21 and p16/Rb pathways and acts as an anti-osteoporotic compound through concurrent activation of osteoblasts and inhibition of osteoclasts.

## 3. Materials and Methods

### 3.1. Cell Preparation and Culture

Primary osteoblast progenitors were isolated from the calvaria of 4-day-old newborn C57BL6 mice (SAMTAKO Bio Korea Co., Ltd., Osan, Republic of Korea) via sequential digestion using 0.25% trypsin (Hyclone, Logan, UT, USA) at 37 °C for 10 min followed by 0.1% collagenase II (Worthington-biochemical Co., Lakewood, NJ, USA) at 37 °C for 30 min [32]. After centrifugation, the cell pellet was suspended in alpha minimum essential medium (α-MEM; Hyclone, Logan, UT, USA) supplemented with 10% (*v*/*v*) fetal bovine serum (FBS; Hyclone) and 1% (*v*/*v*) antibiotic-antimycotic solution (Thermo Fisher Scientific, Inc., Waltham, MA, USA), then seeded into 100 mm cell culture dish and incubated at 37 °C in a humidified atmosphere containing 5% CO_2_.

To isolate osteoclast progenitors [33], bone marrow cells were harvested from the tibia and femur of 7-week-old C57BL6 male mice by flushing the marrow cavity using a 1 mL syringe filled with α-MEM. The cells were filtered through a 40 μm strainer (Falcon Technologies, Inc., Maryland Heights, Missouri, St. Louis, MO, USA), centrifuged, and treated with red blood cell lysis buffer (Sigma-Aldrich, St. Louis, MO, USA) to eliminate red blood cells. The remaining cells were cultured in α-MEM containing 10% (*v*/*v*) FBS, 1% (*v*/*v*) antibiotic-antimycotic solution (Thermo Fisher Scientific, Inc., Waltham, MA, USA), and 5 ng/mL recombinant human macrophage colony-stimulating factor (M-CSF) for 12 h at 37 °C in a 5% CO_2_ humidified atmosphere. Floating cells were centrifuged and further cultured in α-MEM supplemented with 30 ng/mL M-CSF for 2 days to generate osteoclast progenitors. All animal procedures were approved by the Yeungnam University Medical Center (YUMC-AEC2023-027) and carried out in accordance with the Guide for the Care and Use of Laboratory Animals.

### 3.2. Cellular Senescence

Osteoblast (2.0 × 10^4^ cells/well) and osteoclast progenitors (5.0 × 10^4^ cells/well) from passages 3 to 5 were seeded in 48-well plates and treated with 0.2 μM and 1 µM doxorubicin (Sigma-Aldrich, St Louis, MO, USA), respectively, dissolved in dimethyl sulfoxide (DMSO) for 4 h. Following treatment, cells were washed with serum-free α-MEM and exposed to the indicated conditions. Doxorubicin-induced senescent osteoblast and osteoclast progenitors were washed twice with phosphate-buffered saline, fixed in 3.7% paraformaldehyde for 30 min at room temperature, and incubated with SA-β-gal staining solution (0.2 M citric acid/phosphate, pH 6, 5 mM potassium ferrocyanide, 5 mM potassium ferricyanide, 150 mM NaCl, 2 mM MgCl_2_, 0.5 mg/mL X-gal in DMSO) at 37 °C for 16 h and 6 h, respectively. SA-β-gal-positive cells were imaged using a light microscope and counted in four randomly selected regions per well.

### 3.3. Osteoblast and Osteoclast Differentiation

Osteoblast progenitors (2.0 × 10^4^ cells/well) were plated in 48-well plates and differentiated using 4 mM phosphate (Biosolution, Seoul, Republic of Korea) for the indicated durations, with media refreshed after 2 days. To assess differentiation, cells were stained with 40 mM alizarin red S (pH 4.2; Sigma-Aldrich) for 30 min. After imaging the stained plates, cells were dissolved in 10% cetylpyridinium chloride (pH 7.0; Sigma-Aldrich), and absorbance at 550 nm was measured using a microplate reader (Thermo Fisher Scientific, Waltham, MA, USA).

For osteoclast differentiation, osteoclast progenitors (5.0 × 10^4^ cells/well) were cultured in the presence of 30 ng/mL M-CSF and 100 ng/mL recombinant mouse receptor activator of NF-κB ligand (RANKL) for 4 days, with medium replaced on day 2. Cells were stained for tartrate-resistant acid phosphatase (TRAP) using the Leukocyte Acid Phosphatase Staining Kit (Sigma-Aldrich) per the manufacturer’s instructions. TRAP-positive multinucleated cells [TRAP(+) MNCs] with more than three or ten nuclei were counted using light microscopy.

### 3.4. Immunoblot Analysis

Cells were lysed in RIPA buffer [20 mM Tris-HCl, pH 7.5, 150 mM NaCl, 1% Nonidet P-40, 0.5% sodium deoxycholate, 1 mM glycerophosphate acid, 0.1% sodium dodecyl sulfate (SDS), and 1× protease inhibitor cocktail (Roche Holding AG, Basel, Switzerland)]. Lysates were centrifuged, and protein concentrations in the supernatants were measured using a DC protein assay (Bio-Rad, Hercules, CA, USA). Equal amounts of proteins (20 to 30 μg) were separated on 10% or 12% SDS-polyacrylamide gel electrophoresis and transferred to nitrocellulose membranes (Cytiva, Marlborough, MA, USA). Membranes were incubated with primary antibodies: phospho-Rb, p16, p21, p53, alkaline phosphatase (ALP), β-catenin, cathepsin K (Ctsk), NFATc1 or β-actin purchased from SantaCruz (Dallas, TX, USA); NFκB p65 from Cell Signaling Technology (Danvers, MA, USA), and osterix (OSX) and osteopontin (OPN) from Abcam (Cambridge, UK). The appropriate horseradish peroxidase-conjugated secondary antibodies were proved and developed with enhanced chemiluminescence reagents (AbFRONTIER, Seoul, Republic of Korea).

### 3.5. Enzyme-Linked Immunosorbent Assay (ELISA)

SASP factors were measured in culture medium. Conditioned medium was collected by centrifugation at 1400× *g* for 10 min and stored at −80 °C. Concentration of SASP factors (TNF-α, IL-1α, ILβ, IL6, and CCL2) was measured using an ELISA kit (R&D systems; Minneapolis, MN, USA) per the manufacturer’s instructions. Absorbance was read at 550 nm using a microplate reader (Thermo Fisher Scientific).

### 3.6. Ovariectomy and Analysis of Bone Parameter and Histology

Nine-week-old female C57BL6 mice (SAMTAKO) were housed in the laboratory animal facility for one week. At ten weeks of age, mice were anesthetized with 1.25% avertin (Sigma-Aldrich) via intraperitoneal injection and subjected to either sham surgery or bilateral ovariectomy to induce osteoporosis. After recovering for 5 days, cabozantinib (0.25 mg/kg; 500 nM) was administered intraperitoneally every 2 days for 8 weeks.

For body composition analysis, after mice were anesthetized, fat content and bone indices including bone mineral density (BMD) and bone mineral content (BMC) were determined using a high-resolution Dual-energy X-ray Absorptiometry (DXA) cabinet body composition analyzer (iNSiGHT VET DXA, Osteosys, Republic of Korea). In color images, fat regions appear red.

To analyze bone architecture, mice were euthanized and femur and tibiae were excised, fixed in 3.7% formaldehyde for 3 days, and stored in 70% ethanol. Trabecular bone architecture was assessed by high-resolution microcomputed tomography (µCT; Skyscan1276; Skyscan, Aartlesaar, Belgium), and parameters including bone mineral density (BMD), trabecular bone volume per total tissue volume (BV/TV), trabecular bone number (Tb.N), trabecular bone thickness (Tb.Th), and trabecular bone separation (Tb.Sp) were calculated. Cortical bone parameters, such as BMD, area, and thickness, were also assessed. Reconstructed images were obtained using SkyScan NRecon (version 1.7.42).

For histological analysis, fixed femurs were decalcified in 14% neutral-buffered EDTA (Sigma-Aldrich) for 3 weeks and embedded in paraffin. Sagittal sections (5 µm thick) were stained with haematoxylin and eosin (H&E) for osteoblast detection or with TRAP for osteoclast visualization. TRAP-stained sections were further evaluated for bone surface erosion.

### 3.7. Statistical Analysis

All data are presented as mean ± standard deviation (SD) from three independent experiments. Statistical significance was determined using Student’s two-tailed *t*-test for comparisons between two groups, or one-way analysis of variance (ANOVA) for comparisons among three or more groups. A *p*-value < 0.05 was considered statistically significant. Analyses were conducted using GraphPad Prism 8.0.2 software (GraphPad Inc., Software Inc., Boston, MA, USA).

## Data Availability

In this study, the datasets are available on request to the corresponding author.

## Supplementary Materials

The following supporting information can be downloaded at: https://www.mdpi.com/article/10.3390/ijms26157123/s1.
