# Supplementary material for: Cabozantinib, an Anti-Aging Agent, Prevents Bone Loss in Estrogen-Deficient Mice by Suppressing Senescence-Associated Secretory Phenotype Factors
Source: Int J Mol Sci. 2025 Jul 24;26(15):7123. doi: 10.3390/ijms26157123 (PMC12346361; doi:10.3390/ijms26157123)
Supplement: Supplementary file 1 [file ijms-26-07123-s001.zip › ijms-3742915-supplementary.pdf]

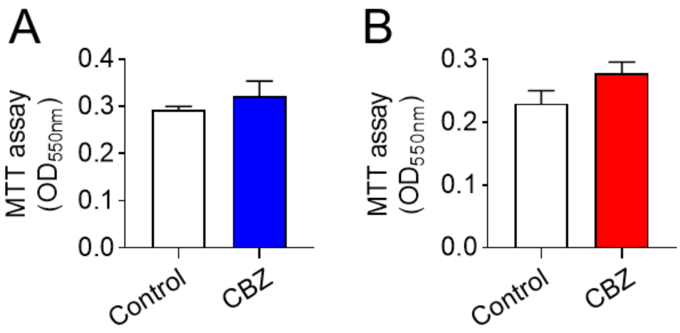

Figure S1. Cytotoxic effects of CBZ on osteoblast and osteoclast progenitors. Osteoblasts (**A**) and osteoclast progenitors (**B**) were treated with doxorubicin for 4 h, followed by culture with or without 100 nM CBZ for 2 days. Cell viability was assessed using the 3-(4,5-dimethylthiazol-2-yl)-2,5-diphenyltetrazolium bromide (MTT) assay. Formazan crystals were solubilized in dimethyl sulfoxide, and the absorbance was measured at 550 nm. Data represent mean ± SD from triplicate experiments.

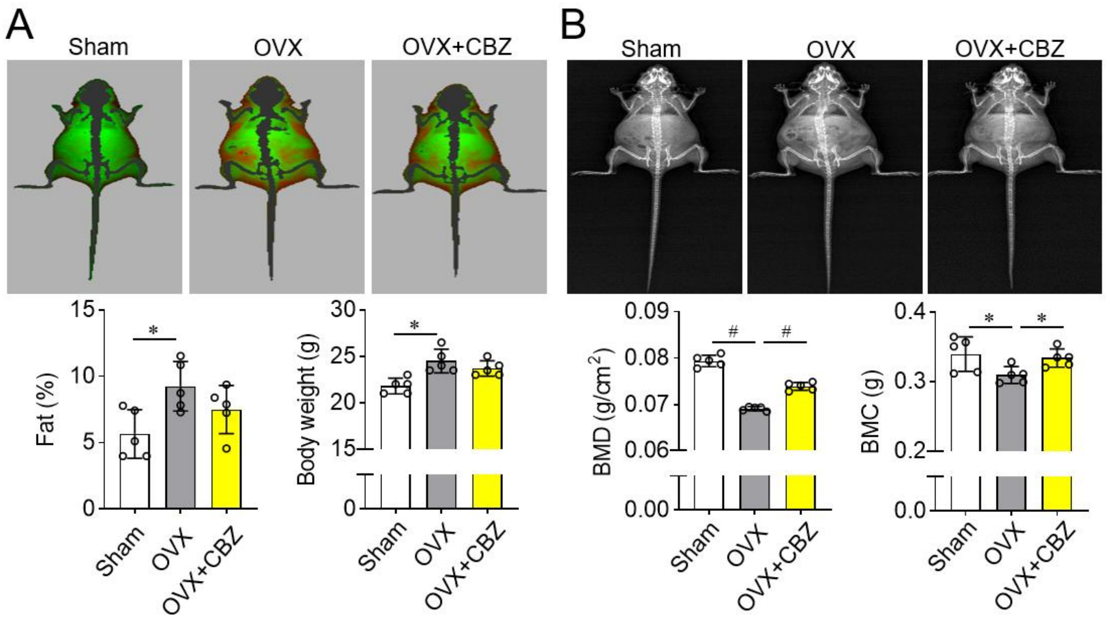

Figure S2. Body composition analysis via DAX. Mice were subjected to either a sham operation or ovariectomy (OVX). CBZ (0.25 mg/kg; 500 nM) was administrated intraperitoneally every two days for 8 weeks. Body fat and weight (**A**), and bone indices [bone mineral density (BMD) and bone mineral content (BMC)] (**B**) were measured by Dual-energy X-ray Absorptiometry (DXA). Red indicates fat in the scanned images (**A**). Data are expressed as mean ± SD (n = 5 per group). $^{*}p < 0.05$; $^{\#}p < 0.01$.

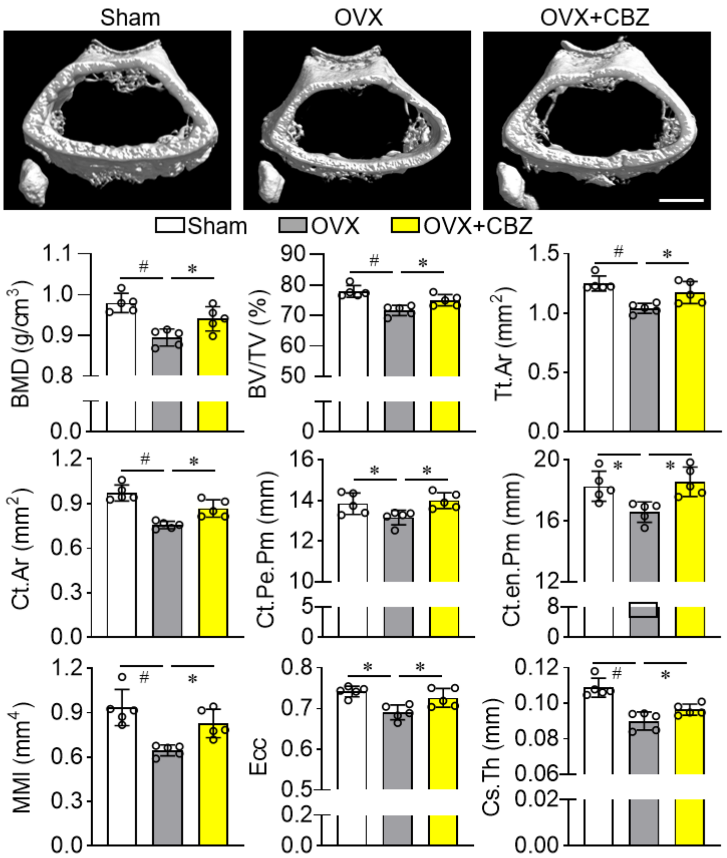

Figure S3. CBZ prevents cortical bone loss in ovariectomized mice. Mice in the sham, OVX, and OVX + CBZ were analyzed (in Figure 4) for cortical bone parameters using μCT. Assessed indices included BMD, bone mineral density; BV/TV, bone volume per total tissue volume; Tt.Ar, total bone area (2D); Ct.Ar, cortical bone area (2D); Ct.Pe.Pm, cortical periosteal perimeter (2D); Ct.en.Pm, cortical endosteal perimeter (2D); MMI, polar moment of inertia (2D); Ecc, mean eccentricity (2D); Cs.Th, cortical cross-sectional thickness (2D). Scale bar, 0.5 mm. Data are presented as mean ± SD (n = 5 per group). $^{*}p < 0.05$; $^{\#}p < 0.01$.

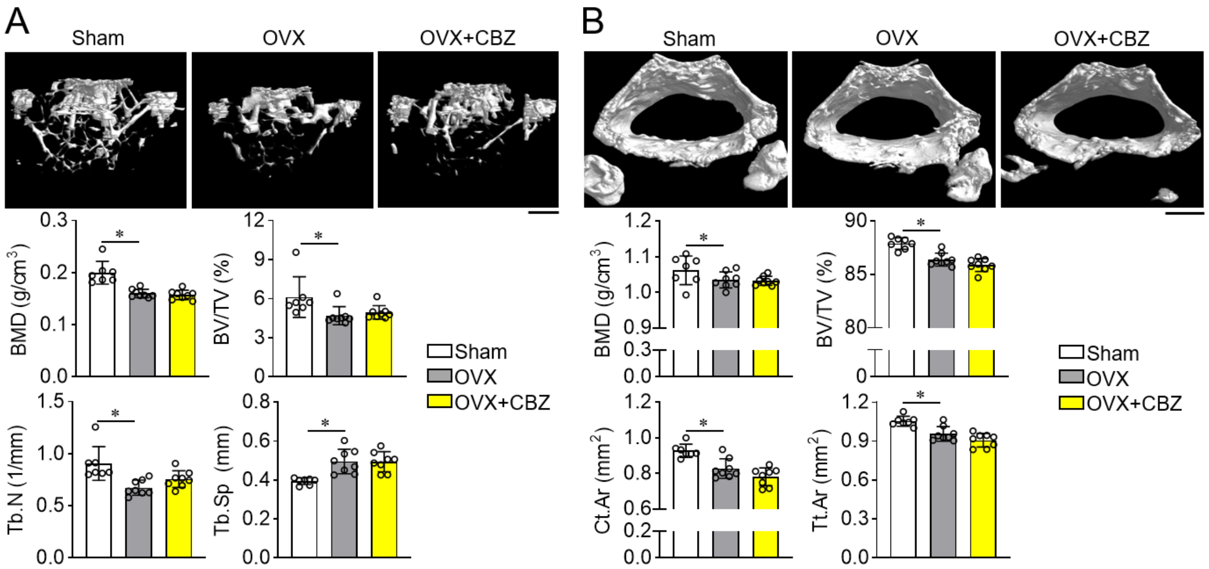

Figure S4. CBZ shows no significant therapeutic effect in delayed treatment of ovariectomized mice. Ten-week-old female C57BL6 mice underwent sham or ovariectomy surgery. Two months post-surgery, ovariectomized mice were divided into OVX and OVX plus CBZ groups. CBZ (0.25 mg/kg; 500 nM) was administrated intraperitoneally every two days for 8 weeks. µCT was used to analyze trabecular (**A**) and cortical bone indices (**B**) in femurs. Scale bar, 0.5 mm. Data are presented as mean ± SD (Sham, n =7; OVX, n =8; OVX + CBZ, n = 8). $^{*}p < 0.05$; $^{\#}p < 0.01$.
